# Resistance to Cry14A family *Bacillus thuringiensis* crystal proteins in *Caenornabditis elegans* operates via the *nhr-31* transcription factor and vacuolar-type ATPase pathway

**DOI:** 10.1371/journal.ppat.1012611

**Published:** 2024-10-18

**Authors:** Youmie Kim, Thanh-Thanh Nguyen, Daniel J. Durning, Takao Ishidate, Ozkan Aydemir, Craig C. Mello, Yan Hu, Theodore W. Kahn, Raffi V. Aroian

**Affiliations:** 1 Program in Molecular Medicine, University of Massachusetts Chan Medical School, Worcester, Massachusetts, United States of America; 2 RNA Therapeutics Institute, University of Massachusetts Chan Medical School, Worcester, Massachusetts, United States of America; 3 Current address: Biology Department, Worcester State University, Worcester, Massachusetts, United States of America; 4 BASF Corporation, Research Triangle Park, North Carolina, United States of America; University of Wisconsin-Madison, UNITED STATES OF AMERICA

## Abstract

*Bacillus thuringiensis* (Bt) has been successfully used commercially for more than 60 years for biocontrol of insect pests. Since 1996, transgenic plants expressing Bt crystal (Cry) proteins have been used commercially to provide protection against insects that predate on corn and cotton. More recently, Bt Cry proteins that target nematodes have been discovered. One of these, Cry14Ab, has been expressed in transgenic soybean plants and found to provide significant protection against the soybean cyst nematode, *Heterodera glycines*. However, to date there has been no description of high-level resistance to any Cry14A family protein in nematodes. Here, we describe forward genetic screens to identify such mutants using the nematode *Caenorhabditis elegans*. Although non-conditional screens failed to identify highly resistant *C*. *elegans*, a conditional (temperature-sensitive) genetic screen identified one mutant, *bre-6(ye123)* (for ***B***t protein ***re***sistant), highly resistant to both Cry14Aa and Cry14Ab. The mutant comes at a high fitness cost, showing significant delays in growth and development and reduced fecundity. *bre-6(ye123)* hermaphrodites are only weakly resistant to copper intoxication, indicating that the mutant is not highly resistant to all insults. Backcrossing—whole genome sequencing was used to identify the gene mutated in *ye123* as the nuclear hormone receptor *nhr-31*. RNAi, DNA rescue, and CRISPR analyses confirm that resistance to Cry14Aa intoxication in *bre-6(ye123)* is due to mutation of *nhr-31* and was renamed *nhr-31(ye123)*. As predicted for a mutation in this gene, *nhr-31(ye123)* animals showed significantly reduced expression of most of the subunits of the *C*. *elegans* vacuolar ATPase (vATPase). Mutants in the vATPase subunits *unc-32* and *vha-7* also show resistance to Cry14Aa and/or Cry14Ab. These data demonstrate that *nhr-31* and the vATPase play a significant role in the intoxication of *C*. *elegans* by Cry14A family proteins, that reduction in vATPase levels result in high resistance to Cry14A family proteins, and that such resistance comes at a high fitness cost. Based on the relative difficulty of finding resistant mutants and the fitness cost associated with the vATPase pathway, our data suggest that transgenic Cry14Ab plants may hold up well to resistance by nematode parasites.

## Author summary

The bacterium *Bacillus thuringiensis* (Bt) is the most common biologically-produced insecticide used in agriculture. The main insect-killing components of Bt are crystal (Cry) proteins. Cry proteins that kill nematode (roundworm) pests have also been identified. One of these, Cry14Ab, is being developed to control plant-parasitic nematodes, which are highly-damaging pests of agriculture. Thus, understanding how nematodes become resistance to Cry14Ab is important. Using the free-living nematode *Caenorhabditis elegans*, we discovered a single mutant strain highly resistant to Cry14A family proteins. This mutant was rare and has compromised health in the absence of Cry protein (*i*.*e*., it grows slowly and has low brood size). We identified the gene altered in this mutant as *nhr-31*, a gene that is involved in making a large cellular protein machine that pumps protons across membranes. We found that reduction of function of other components of this proton-pumping machine also result in resistance to Cry14A family proteins. Taken together, our data suggest that a major pathway involved in resistance to Cry14A family proteins is reduction of function of a large cellular proton pump machine that comes at a high fitness cost to nematodes.

## Introduction

*Bacillus thuringiensis* (Bt) is a common Gram-positive soil bacterium found around the world that produces pesticidal proteins [1]. The most well studied are crystalline (Cry) proteins, which accumulate in parasporal crystal inclusions during sporulation [1]. Each Cry protein kills a narrow set of target species that include major insect pests and nematodes [2,3]. Cry proteins are biodegradable and innocuous for plants, vertebrates, and humans [4]. Because of these properties, Cry proteins have been used for decades to kill insect vectors of disease as well as crop pests in conventional and organic farming[1], accounting for ~90% of all microbial biopesticides marketed worldwide [5]. Cry proteins are produced in transgenic crops such as corn, cotton, and soybean that were planted on a cumulative total of 1.5 billion hectares from 1996 to 2022 [6,7]. More than a dozen Cry proteins, including those expressed in transgenic crops, have been studied extensively and approved as safe for human consumption by the EPA and FDA [8–10]. In acute oral toxicity testing of mice with doses reaching 3000 to 5000 mg of Cry protein per kg body weight, no significant effects were seen in the test animals [8,11].

Hundreds of Cry proteins in >50 different families have been characterized [12,13]. We found that some Cry proteins (*e*.*g*., Cry5Ba, Cry21Aa, Cry14Aa), related by sequence and structure to those used to combat insects, can kill nematodes [14,15]. Cry5Ba has been studied extensively against human and animal gastrointestinal nematode (GIN) parasites and when administered orally is effective *in vivo* against a wide range of GIN infections in animals including against human hookworm (*Ancylostoma ceylanicum*, *Necator americanus*) infections in rodents, hookworm (*Ancylostoma caninum*) infections in dogs, large roundworm (*Ascaris/Parascaris*) infections in pigs, horses, and mice, and barber’s pole worm (*Haemonchus contortus*) infections in sheep [16–19]. Cry5Ba protein was also expressed in transgenic tomato roots and provided control over infection by the root-knot plant-parasitic nematode (PPN) *Meloidogyne incognita*, significantly impairing the ability of *M*. *incognita* to form galls, egg masses, and eggs [20]. This result suggested that transgenic plants expressing a nematode-active Cry protein might provide protection against endoparasitic PPNs.

With this goal in mind, Cry14Ab (83% and 30% amino acid identity to Cry14Aa and Cry5Ba in active domain, respectively) was expressed in transgenic soybean plants [21]. Both in greenhouse trials and field trials in Iowa, transgenic Cry14Ab soybean plants significantly impaired the reproduction of by the PPN soybean cyst nematode (*Heterodera glycines*) (60% reduction in field trials at end-of-season [21]). *H*. *glycines* is one of the most important pathogens of soybeans worldwide, and the main source of soybean yield loss to disease in the United States, with yield losses exceeding one billion dollars [22]. Transgenic Cry14Ab soybean plants, which have received regulatory approval from the US Environmental Protection Agency (EPA) and Food and Drug Administration (FDA), can fill an important gap in providing protection against *H*. *glycines* [23,24].

To date, however, whether or not nematodes could develop high-level resistance to Cry14A family proteins and, if so, via what pathways, was not known. Since this question is critically important with regards to deployment of transgenic crops expressing Cry14A family proteins on a large scale, here we describe the isolation, identification, and characterization of a newly identified *Caenorhabditis elegans* strain and pathway that mutates to resistant to Cry14A family proteins (Cry14Aa and Cry14Ab), albeit with a high fitness cost.

## Results

### Identification of *bre-6(ye123)* highly resistant to Cry14Aa and Cry14Ab

We previously reported that *C*. *elegans* Cry5Ba glycosphingolipid receptor mutants (*bre-2(ye31)*, *bre-3(ye28)*, *bre-4(ye13)*, and *bre-5(ye17); bre* stands for ***B***t-protein ***re***sistant), which were found by screening for resistance to Cry5Ba and that are highly resistant to Cry5Ba, have low-to-moderate resistance to Cry14Aa [25,26]. Here, we compared the resistance of these same glycosphingolipid mutants to both Cry14Aa and Cry14Ab (S1 Table). We confirmed that these *bre* glycosphingolipid mutants showed relatively low levels of resistance against Cry14Aa, and we found that they showed even lower levels of resistance to Cry14Ab.

To find mutants with higher levels of resistance, we screened large numbers of mutagenized N2 wild-type hermaphrodites (~794,000 F2) for resistance to Cry14Aa expressed in *Escherichia coli* (see Materials and Methods). We found two novel mutations in *bre-3*, which were not strongly resistant. We therefore hypothesized that high-level resistance may either (1) come at a high fitness cost or (2) requires mutation of multiple genes simultaneously. Either of these would make identification using a simple forward genetic screen difficult.

**Fig 1:**
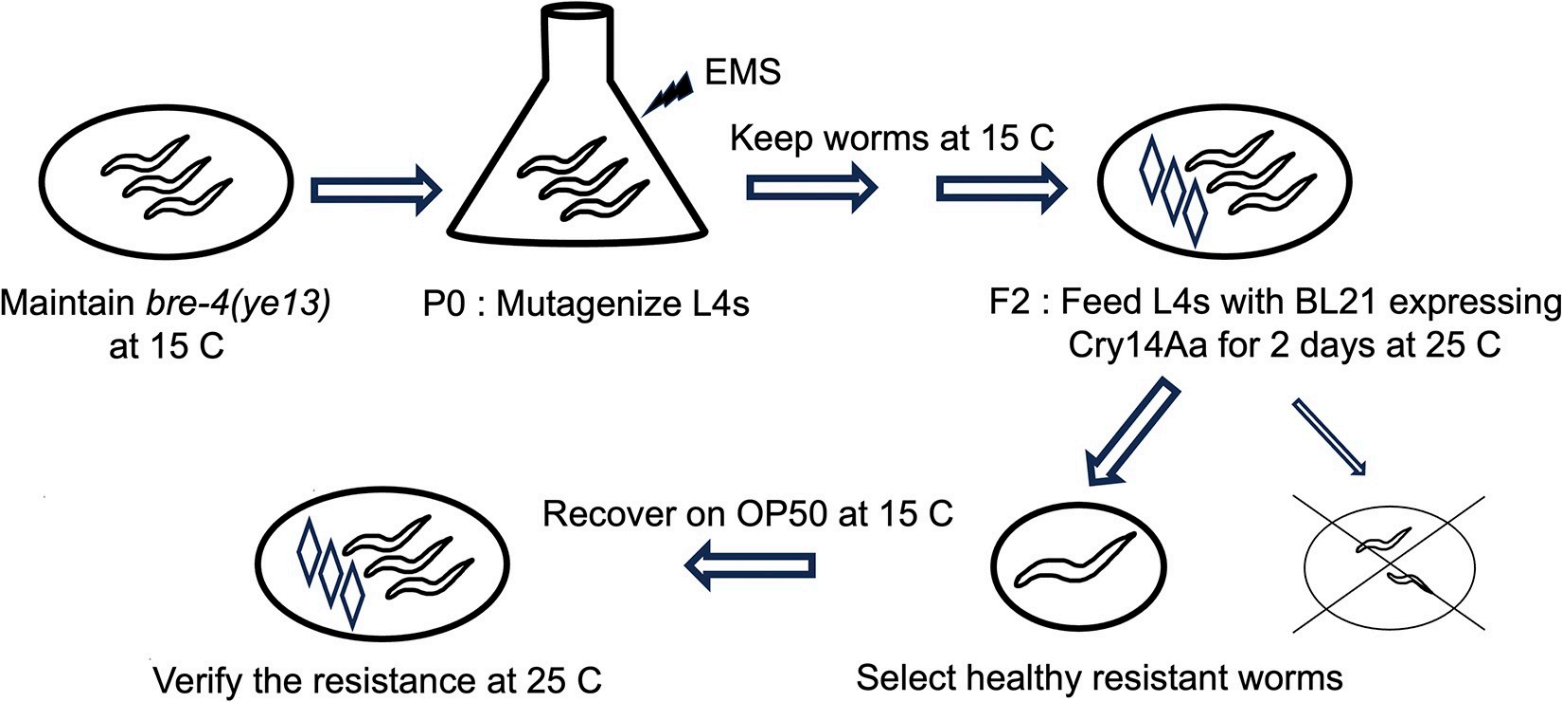
Schematic of condition resistance screen for Cry14Aa-resistant *C*. *elegans*.

To address hypothesis 1 (fitness cost), we modified the *C*. *elegans* screen from a non-conditional to a temperature-conditional screen (Fig 1). Our aim was to identify a temperature-sensitive mutant that could be maintained at the permissive temperature (15° C) but shifted to a non-permissive temperature (25° C) for resistance testing (at a time when function is no longer are required for viability, fertility…). To prevent the chance of finding mutation in the known glycosphingolipid receptor pathway, the screen was carried out in a *bre-4(ye13)* background. After extensive screening using Cry14Aa (~175,000 F2), we identified a single resistant mutant, *bre-6(ye123)*. *bre-6(ye123)* is resistant to both Cry14Aa and Cry14Ab and is characterized in detail below.

**Fig 2:**
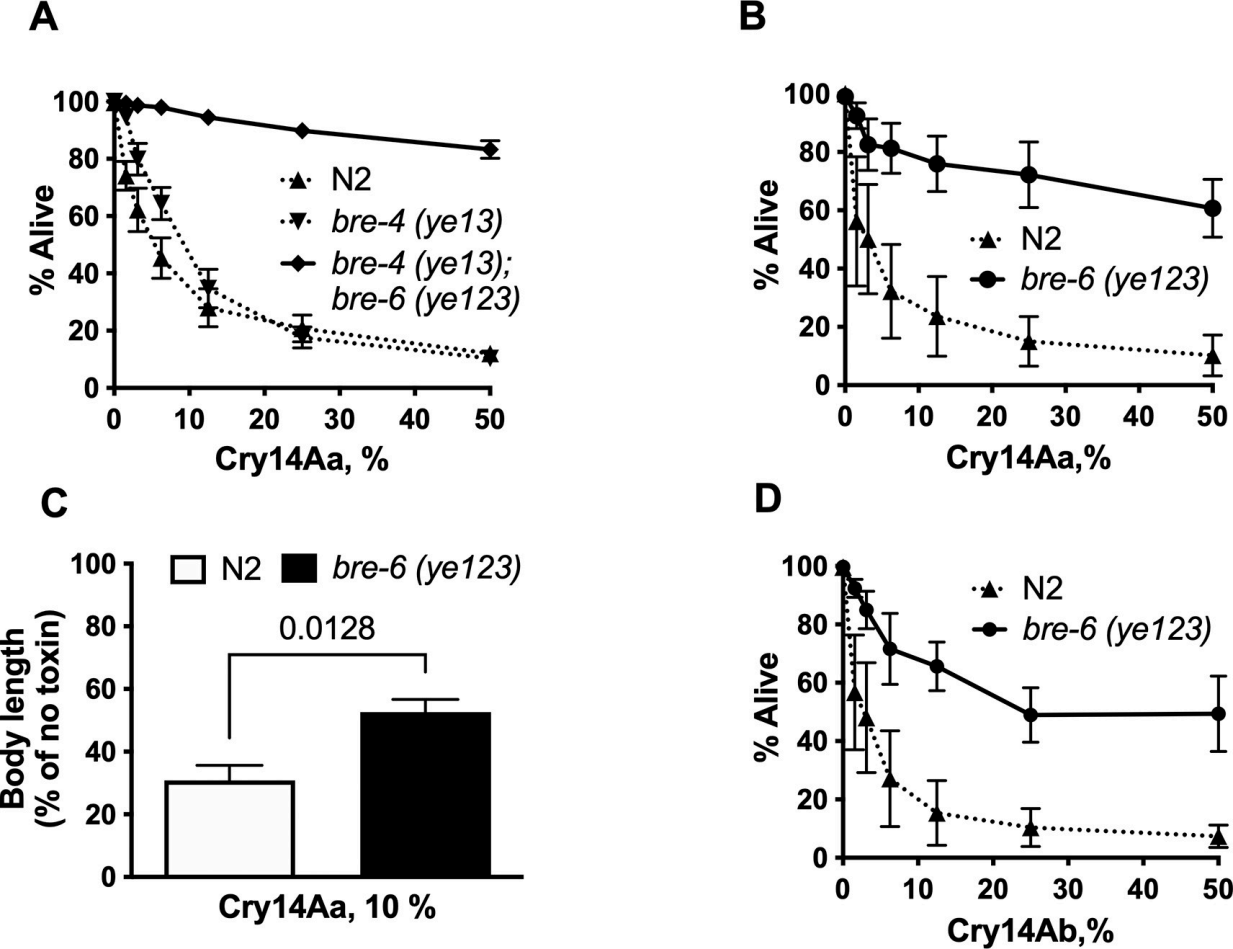
Quantitation of *bre-6(ye123)* resistance to Cry14A proteins. A. Resistance of outcrossed *bre-4 (ye13);bre-6 (ye123)* to Cry14Aa expressed in *E*. *coli* relative to N2 and *bre-4(ye13)* based on viability (dose-response mortality assay in 48-well format). Note the slight resistance of *bre-4(ye13)* alone to Cry14Aa relative to N2 wild-type. B. Resistance of *bre-6(ye123)* to Cry14Aa relative to N2 based on viability (dose-response mortality assay). C. Resistance of *bre-6(ye123)* to Cry14Aa relative to N2 based on growth (body length over time relative to no-toxin control). Relative to no-toxin control, *bre-6(ye123*) animals grew to a longer length than N2 animals for 3 days at 25°C. P calculated one-tailed Student’s t test. D. Resistance of *bre-6(ye123)* to Cry14Ab relative to N2 based on viability (dose-response mortality assay). The error bars denote standard error of the means based on data from three independent experiments. To achieve the variation in doses (abscissa), OD normalized bacteria were diluted with vector control such that 0% is all vector only *E*. *coli* and 100% is undiluted Cry14A-expressing *E*. *coli* (see Materials and Methods).

*bre-6(ye123)* was originally isolated in the *bre-4(ye13)* background. Relative to wild-type N2 and *bre-4(ye13)* mutants alone, *bre-4(ye13);bre-6(ye123)* double mutant hermaphrodites are resistant to Cry14Aa expressed in *E*. *coli* both qualitatively (S1 Fig) and quantitatively (Fig 2A). Upon further outcrossing and removal of the *bre-4(ye13)* allele from the double mutant, *bre-6(ye123)* hermaphrodites were still highly resistant to Cry14Aa as measured both by survival (Fig 2B) and growth (Fig 2C). *bre-6(ye123)* hermaphrodites were also highly resistant to Cry14Ab expressed in *E*. *coli* (Fig 2D). Whereas virtually all N2 are dead at the highest dose tested (Fig 1B and 1D), 50% lethality was reached with neither Cry14Aa nor Cry14Ab at this dose, making it not possible to calculate a lethal dose 50% (LD_50_).

### *bre-6(ye123)* hermaphrodites have a significantly reduced fitness

**Fig 3:**
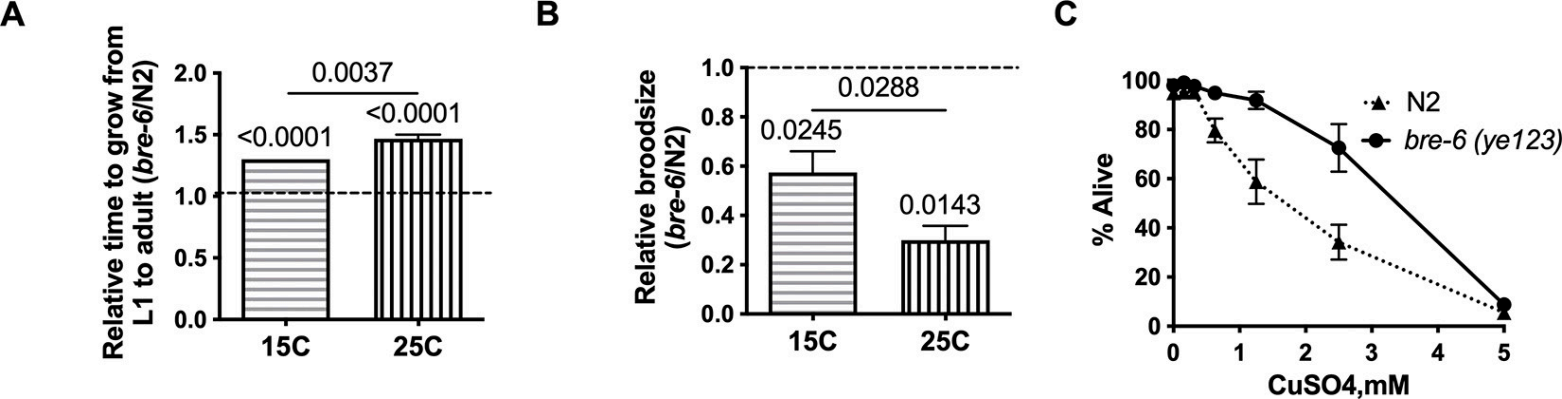
Characterization of fitness cost of *bre-6*(*ye123*). (A) The number of hours from the L1 stage to egg-bearing adult at both 15°C and 25°C was measured for N2 and *bre-6(ye123)* and then normalized to N2 for each temperature (average of three independent experiments). P value above each bar represents the comparison of each to the value 1 (N2; one sample Student’s t test). The relative time to growth at 15°C = 1.3 and at 25°C = 1.47 (P = 0.0037 comparing *bre-6(ye123)* at 25° to *bre-6(ye123)* at 15°C, one-tailed Student’s t test). (B) The brood size of individual hermaphrodites starting at the L4 stage and then allowed to produce progeny for 72 hr. at each temperature was recorded (n = 4 hermaphrodites/experiment repeated 3 times) and then normalized to N2 for each temperature. Actual values are: 15°C: N2(157), *bre-6(ye123)*(88); 25°C: N2(58.5), *bre-6(ye123)*(18.1).). P value above each bar represents the comparison of each to the value 1 (N2; one sample Student’s t test). The relative brood size at 15°C = 0.57 and at 25°C = 0.3 (P = 0.0288 comparing *bre-6(ye123)* at 25° to *bre-6(ye123)* at 15°C, one-tailed Student’s t test). (C) The viability of wild-type and *bre-6(ye123)* mutant animals at various doses of the heavy metal copper at 25° C (n = 4).

Since high resistance to Cry14A-family Cry proteins via *bre-6(ye123)* was found using a conditional genetic screen, we hypothesized that the mutant allele came with a fitness cost that would be higher at the non-permissive temperature and that would have made it more difficult to isolate in a non-conditional screen. To test this hypothesis, we measured the growth rate of *bre-6(ye123)* hermaphrodites from the L1 to the egg-bearing young adult stage relative to wild-type N2 animals both at 15°C and 25°C (Fig 3A). At both 15°C and 25°C, growth rate of the mutant relative to N2 wild type is significantly delayed by 30% and 47% respectively. In addition, the growth rate relative to N2 wild type is significantly more delayed at 25°C than 15°C (P = 0.0037), indicating that the fitness cost at the non-permissive (resistant) temperature is higher.

We also measured the fecundity *of bre-6(ye123)* hermaphrodites relative to N2 wild-type at both 15°C and 25°C. *bre-6(ye123)* hermaphrodites have a significantly reduced total brood size relative to N2 wild type (Fig 3B). At 15°C, fecundity was reduced by 43%, whereas at 25°C fecundity was reduced by 70%. Moreover, the relative fecundity at 25°C was significantly lower than the fecundity at 15°C (P = 0.029), again indicating that the fitness cost at the non-permissive (resistant) temperature is higher.

We next assayed the response of *bre-6* to a non-related stressor to determine if strong Cry14A resistance in *bre-6(ye123)* might carryover to another insult. We performed dose-response assays of *bre-6(ye123)* on the heavy metal copper at 25°C, a toxic agent to *C*. *elegans* that is unrelated to Cry proteins but kills with similar kinetics [27–30]. We found that *bre-6(ye123)* hermaphrodites were slightly resistant to copper 25°C (Fig 3C; lethal concentration 50% or LC_50_ for *bre-6(ye123)* was 1.8X higher than the LC_50_ for N2). Resistance to copper was far weaker than resistance to Cry14A family proteins (compare Fig 2B and 2D to Fig 3C). Taken together, these data indicate that *bre-6(ye123)* hermaphrodites have a higher fitness cost relative to N2 wild-type at 25°C relative to 15°C and that the mutant is not highly resistant to all insults.

### *ye123* resistance to Cry14A is due to mutation in the *nhr-31* gene

**Fig 4:**
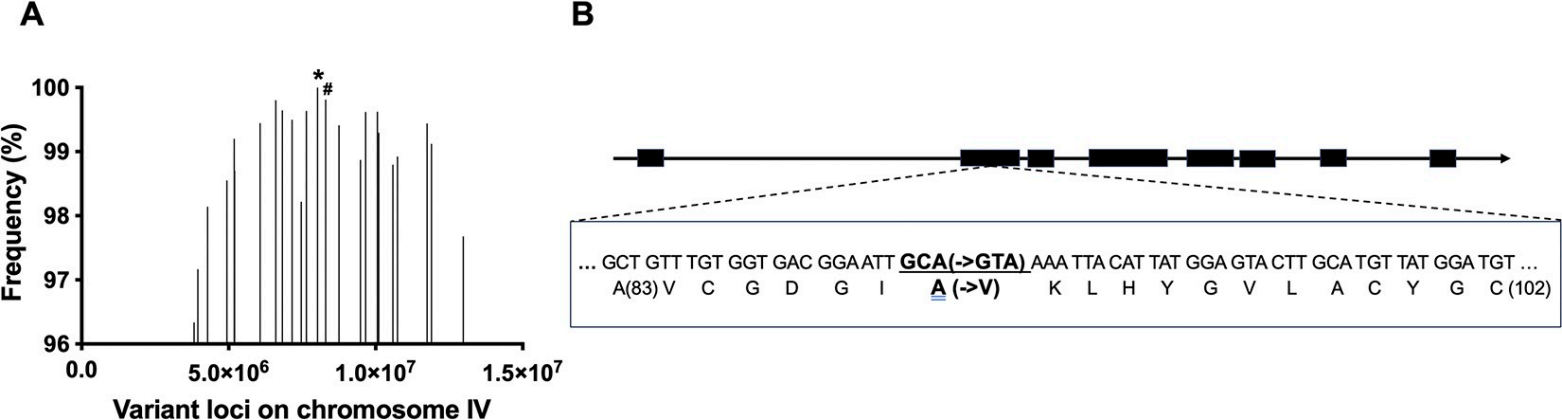
Mapping and sequencing of *bre-6(ye123)*. A. Frequencies and loci of variants on chromosome IV of *bre-6(ye123)* by whole genomic sequencing. The variant in *nhr-31* (indicated by *) occurred at 100% frequency, and the locus centered among variants loci having over 96% of frequencies. A commonly occurring variant in *vha-5* (indicated by #), which is in the same pathway, was also found (see Discussion). B. Alteration of *nhr-31 in bre-6(ye123)*. The mutation in DNA sequence found in the *nhr-31* variant alters amino acid 90 from alanine to valine, which is found in one of two zinc finger DNA binding domains encoded in *nhr-31*. Black boxes are exons.

To identify the gene mutated in *bre-6(ye123)* associated with resistance to Cry14A family proteins, we used the variant discovery method utilizing backcrossing and whole genomic sequencing [31]. Briefly, we backcrossed the mutant to the parent strain, re-isolated homozygous resistant hermaphrodites, and, using whole-genomic sequencing, compared nucleotide variants consistently retained in resistant hermaphrodites (generated by mutagenesis) but not present in the parent strain (see Materials and Methods for details). Based on these data, we found that resistance mapped to a small region around chromosome IV (+3.6), within which a single nucleotide change in the nuclear hormone receptor 31 (*nhr-31)* gene arose at 100% frequency (Fig 4A). *nhr-31* encodes a *C*. *elegans* ortholog of mammalian hepatocyte nuclear factor 4α (HNF-4α) and has been identified as a putative transcriptional activator of multiple subunits of the vacuolar-type (v) ATPase, a pump that hydrolyzes ATP to transport protons across cellular membranes [32]. The mutation found in *ye123* is found in one of the zinc finger DNA binding domains of NHR-31 (Fig 4B) and is conserved in the two closest zinc fingers in *C*. *elegans*, namely those in *nhr-7* and *nhr-119*.

**Fig 5:**
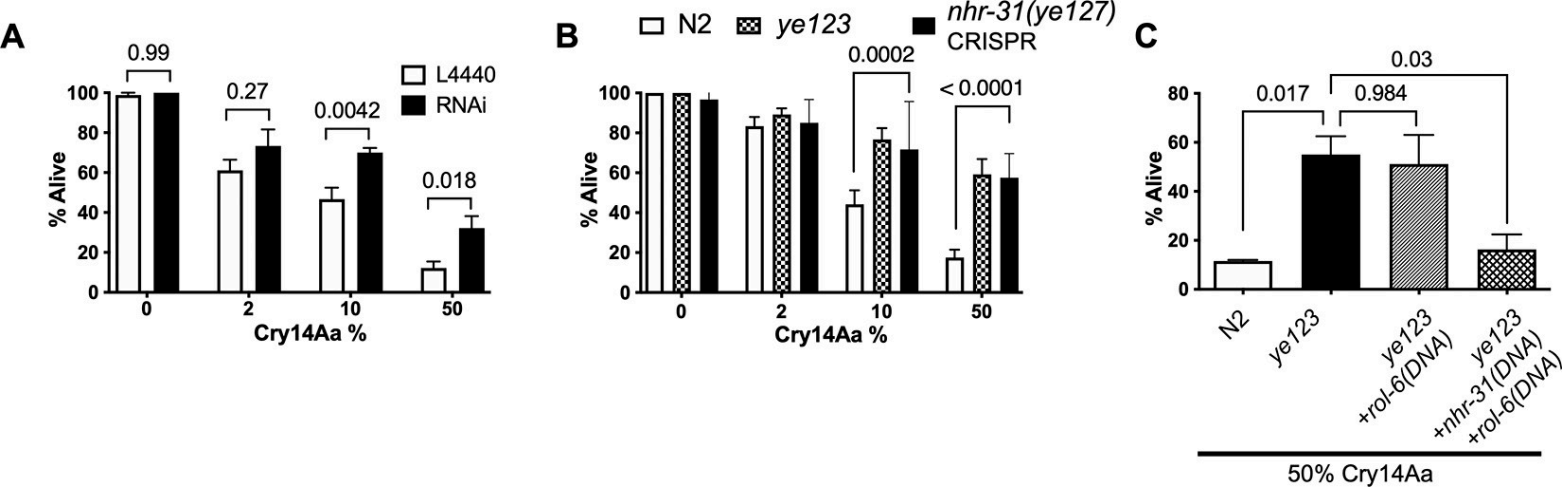
*ye123* is a mutant of *nhr-31*. (A) RNAi of *nhr-31* recapitulates resistance to Cry14Aa. Open bar–N2 fed L4440 empty vector (RNAi control); Filled bar—N2 treated *with nhr-31*. P values from two-way analysis of variance (ANOVA) with Sidak’s multiple comparison test. (B) CRISPR recreation of the *ye123* point mutant in N2 (designated *nhr-31(ye127)*) recreates resistance seen with *ye123*. P values from two-way ANOVA as in panel A. (C) Cry14Aa resistance of *ye123* was rescued with *nhr-31* wild-type extrachromosomal array. *ye123*+*rol-6(DNA)* is *ye123* transformed with *rol-6* gene; *ye123*+*nhr-31(DNA)+rol-6(DNA)* is *ye123* transformed with wild-type *nhr-31* gene + *rol-6* gene. P values by one-way ANOVA and Tukey’s multiple comparison test. For panel A, n = 90; for panel B, n = 120; for panel C, n = 30.

To confirm that reduction of *nhr-31* gives rise to Cry14A resistance, we performed RNA interference (RNAi) experiments (Fig 5A). Knock-down of *nhr-31* (confirmed by real-time PCR) resulted in resistance to Cry14Aa (Fig 5A). We also performed CRISPR (Clustered Regularly Interspaced Short Palindromic Repeats)-Cas9 genome editing to re-create the same *nhr-31* mutation present in *ye123*. As shown, the level or resistance seen to Cry14Aa in this CRISPR allele (*nhr-31(ye127)*) was similar to the original mutant (Fig 5B). The slow growth and small size of *ye123* were also recreated with the *nhr-31(ye127)* CRISPR mutant.

Conversely, we asked if transformation of the *ye123* mutant with a wild-type copy of *nhr-31* could be rescued to wild-type Cry14Aa susceptibility. *ye123* hermaphrodites were transformed using microinjection with a ~5 kb genomic PCR construct that includes the entire gene with introns and two kilobases of promoter and one kilobase of 3’ untranslated region (UTR). As shown in Fig 5C, the wild-type *nhr-31* gene rescues *ye123* hermaphrodites back to sensitivity. Taken together, our RNAi, CRISPR, and rescue data demonstrate that resistance associated with *ye123* mutant animals to Cry14 family proteins is due to mutation in the *nhr-31* gene. For the remainder of the paper, we will refer to *ye123* as *nhr-31(ye123)* and note that *bre-6* has been approved as an “Other Name” for *nhr-31*.

### The vATPase pathway is affected in the *nhr-31* mutant and can give rise to Cry14A resistance

**Fig 6:**
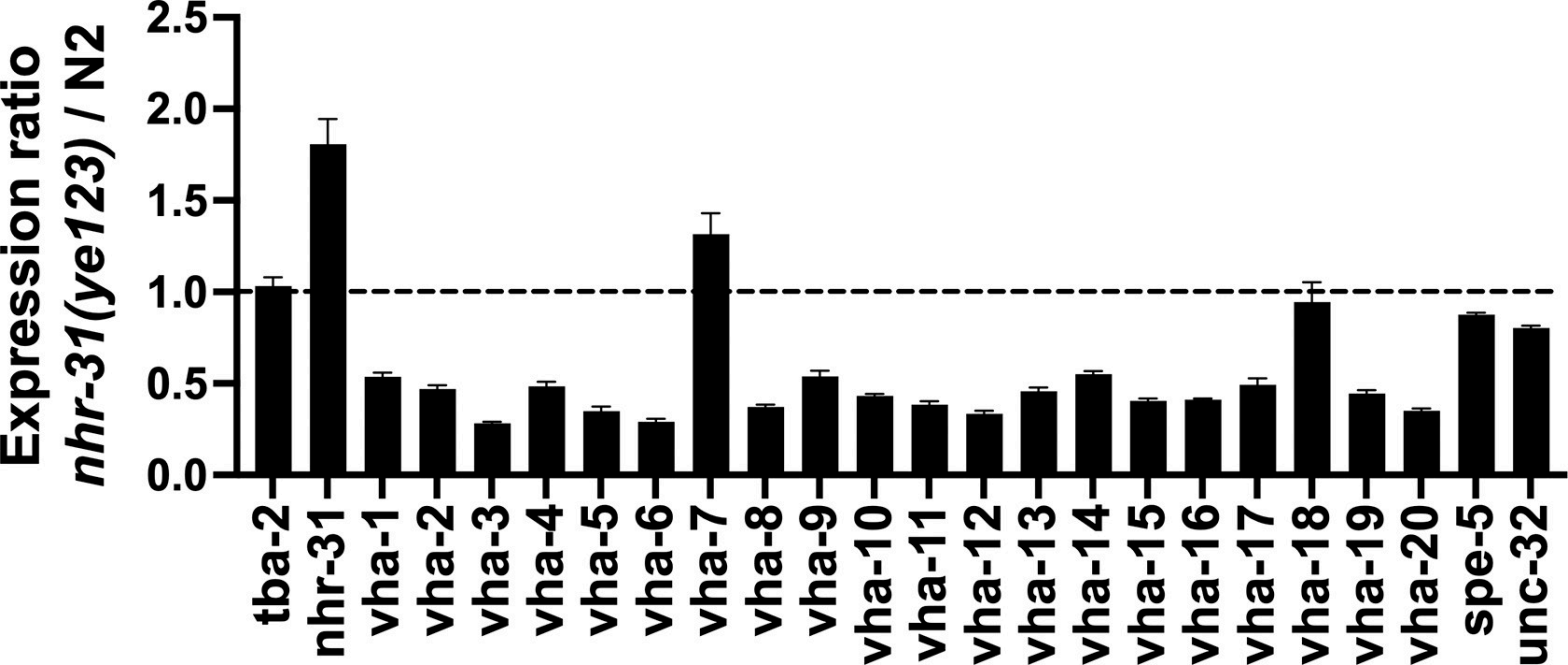
mRNA expression levels of V-ATPases in *nhr-31(ye123)* compared to N2. N2 and *nhr-31(ye123)* nematodes were harvested after 4 hr. of incubation at 25°C on the plates with BL21 bacteria harboring empty vector pRSF. The mRNA expression levels were determined by RNAseq (three independent repeats). The expression in *tba-2* and *vha-18* were statistically unchanged from *nhr-31(ye123)* and N2 animals at q<0.05 (q = 0.14, = 0.15 respectively).

Since *nhr-31* is a known transcriptional regulator of many subunits of the vATPase [32], we hypothesized that *nhr-31(ye123)* mutants should have altered mRNA levels of vATPase genes. We performed RNA-seq, comparing *nhr-31(ye123)* L4 hermaphrodites to N2 wild-type hermaphrodites and studied the expression of *vha* genes (Fig 6). As shown, many *vha* genes are down-regulated in the *nhr-31(ye123)* mutant, consistent with reduction of *nhr-31* function. Qualitatively, the changes seen are very similar to those reported for *nhr-31* RNAi [32]. Of particular note is the lower dependence of *vha-7* and *unc-32* transcript levels on *nhr-31/bre-6* function, which has been previously seen (Fig 6; [32]).

**Fig 7:**
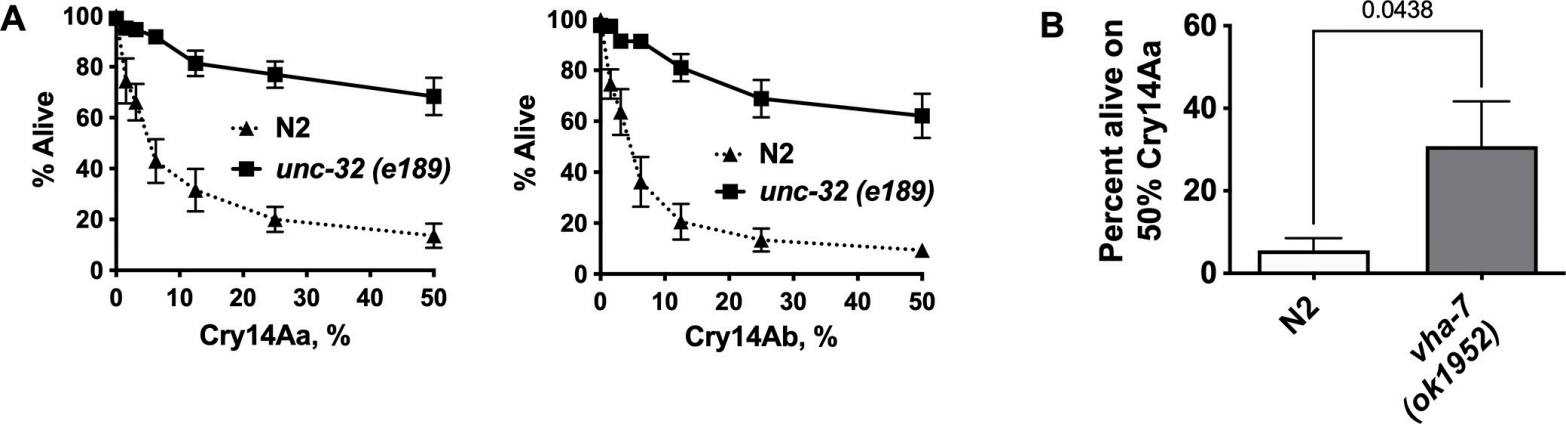
Resistance of other mutants in the vATPase to Cry14A family proteins. (A) Quantitation of *unc-32(e189)* resistance to Cry14A proteins (left Cry14Aa, right Cry14Ab) based on viability (dose-response mortality assay). (B) Quantitation of *vha-7(ok1952)* mutants to 50% Cry14Aa. P value based on students one-tailed T test. Each graph represents the average of three independent experiments.

Taken together, our data suggest that resistance of *nhr-31(ye123)* was caused by reduction of vATPase function. To test this hypothesis independent of *nhr-31(ye123)*, we asked whether or not mutations directly in vacuolar subunits, namely *vha-7(ok1952)* and *unc-32(e189)* hermaphrodites, also result in resistance to Cry14A family proteins. *vha-7* and *unc-32* both encode vATPase a subunits that also have a relatively lower level of reliance on transcription by *nhr-31* [32] (Fig 6) (as with *nhr-31(ye123)* hermaphrodites, *unc-32(e189)* and *vha-7(ok1952)* hermaphrodites were slow growing). As shown in dose-response assays (Fig 7A), reduction of vATPase function via the allele *unc-32(e189)* resulted in high levels of resistance to both Cry14Aa and Cry14Ab. Reduction of vATPase function via the allele *vha-7(ok1952)* also resulted in resistance to Cry14Aa (Fig 7B).

## Discussion

Direct screening for *C*. *elegans* resistant to Cry14A family proteins failed to produce any *C*. *elegans* mutant with high resistance whereas conditional screening was successful in identifying *bre-6(ye123)*, renamed *nhr-31(ye123)*. At 25° C, *nhr-31(ye123)* hermaphrodites are highly resistant to both Cry14Aa and Cry14Ab proteins. The level of resistance against Cry14A is much higher than seen with the Cry5Ba-generated glycosphingolipid receptor mutants against Cry14A. Interestingly, the original un-backcrossed *nhr-31(ye123);bre-4(ye13)* animal contained a single nucleotide polymorphism (SNP) in the vATPase subunit *vha-5*, which is closely linked to *nhr-31*. This polymorphism was removed during outcrossing and only appears in the data in Figs 2A and 4A. A comparison of the level of resistance in Fig 2A vs 2B indicates that the *vha-5* SNP might have contributed a small level of resistance during initial isolation and likely contributed initially to the identification of the mutant during the screening process.

At both 15°C and 25°C, *nhr-31(ye123)* hermaphrodites have significant fitness costs as assessed by slow growth rate and reduced brood size, with the fitness cost much less pronounced at 15°C, which may have allowed for the conditional screen to work. To date, we have not been able to assess to what degree *nhr-31(ye123)* animals are resistant at 15°C because, for *C*. *elegans*, Cry proteins are much less potent at that temperature. High fitness costs have been previously associated with mutations in this pathway in *C*. *elegans*, and the vATPase is involved in important and essential processes in many organisms [32,33]. Indeed, knock-down of vATPase function is lethal to rootworm beetles feeding on transgenic corn plants expressing double-stranded RNA for vATPase subunit A, presenting a means for biological control of this pest [34].

Genetic backcrossing, whole-genome sequencing, RNAi, CRISPR, and rescue data all converge to demonstrate that the resistance phenotype of *nhr-31(ye123)* is attributed to mutation in the *nhr-31* gene, most well noted for its role in transcriptional regulation of the vATPase [32]. Consistent with this function of *nhr-31*, we find that transcript levels of 18 subunits of the vATPase complex are down regulated in the *nhr-31(ye123)* mutant. Our data indicate that resistance to Cry14A family proteins is caused by reduction of function of the vATPase. In addition to the *nhr-31(ye123)* phenotype, reduction of function of two a subunits, *unc-32* and *vha-7*, also result in resistance to Cry14A family proteins. We note that resistance alleles in these genes were not isolated in our screen. Perhaps these could have been identified in a larger screen or with a different selection or are not amenable to isolation in the conditional screen used here.

This report is not the first to report that reduction of a *vha* mutant can give rise to *C*. *elegans* resistant to a nematicidal Bt protein. A *vha-12* mutant was found to be resistant to the Bt protein App6A (unrelated to Cry14A [14,35]), which was attributed to defects in cell necrosis [36]. It is not clear if this result is relevant here since in the same study *vha-12* mutant hermaphrodites were not resistant to Cry5Ba [36], which is related to Cry14A [14]. The mechanism by which *nhr-31(ye123)* mutants and vATPase mutants are resistant to Cry14A resistance is not known. We speculate it might be relevant to production of a receptor or to diminishing of a cytotoxic cellular response mediated by the vATPase (*e*.*g*., the movement of protons by the vATPase might contribute to intoxication). In agreement with our results, data from two previous studies indicated that reduction of function of vATPase function may result in reduced insecticidal Cry protein intoxication in insects [37,38].

A main aim of the study was to understand the pathways that might give rise to Cry14A resistance and how well Cry14A transgenic crops might hold up to selective pressure in nematodes. We did not directly extend our results to plant-parasitic nematodes. Nonetheless, *C*. *elegans* has proven to be pivotal for studying the mechanism of action of virtually all anthelmintics in use (*e*.*g*., benzimidazoles, ivermectin, levamisole, pyrantel, emodepside,…) [39,40], as well as compounds toxic to PPNs [41,42], indicating that the data here are likely relevant for understanding such pathways with Cry14A nematicides. Our data are also suggestive that Cry14A-crops may hold up well to selective pressure. First, extensive non-conditional screening against Cry14A family proteins did not yield highly resistant mutants, in contrast to similar *C*. *elegans* forward genetic screens against the three main classes of anthelmintics in use today in which resistant nematodes were readily isolated [43]. Second, the known glycosphingolipid mutants show ~10-fold resistance to Cry14Aa and even less to Cry14Ab. Third, conditional screening of 175,000 F2 progeny against Cry14Aa gave rise to one mutant, *nhr-31(ye123)*, that is significantly resistant to both Cry14Aa and Cry14Ab. Thus, resistance is not a common phenotype in a forward genetic screen. Fourth, *nhr-31(ye123)* animals have significant fitness costs in terms of reproduction and growth rate, as is generally associated with mutations in the vATPase pathway [32–34]. Fifth, their ability to resist other stressors, here the heavy metal copper, was only slightly enhanced. Taken together, these data are suggestive that the transgenic Cry14A-crops may hold up well to selective pressure by parasitic nematodes.

## Materials and methods

### Maintenance of *C*. *elegans* and synchronization and outcrossing

Standard *C*. *elegans* techniques were used as described [44]. The strains used in this study include *C*. *elegans* Bristol N2 strain wild-type nematodes, PD4792(mIs11 [myo-2p::GFP + pes-10p::GFP + gut-promoter::GFP]), *unc-32(e189)*, and *vha-7(ok1952)*, all provided by the *Caenorhabditis* Genetics Center. We constructed a double *bre-4*(*ye13*):*GFP* strain by mating *bre-4*(*ye13*) hermaphrodite with PD4792 males, selecting GFP F1s, selfing, selecting GFP F2s, and confirming homozygosity for *bre-4*(*ye13*) allele using PCR and for GFP by noting all progeny were GFP positive. All were cultured using standard techniques including the use of *Escherichia coli* strain OP50 as a standard food source on NG plates [44]. All *C*. *elegans* strains were maintained at 15°C. *C*. *elegans* was synchronized by hypochlorite treatment. Hermaphrodites for toxicity assays were achieved by seeding L1 animals on OP50 and growing at 15°C until reaching the fourth larval (L4) stage.

### Expression of Cry proteins

Cry14Aa and Cry14Ab encoding DNA in the pRSF vector were provided by BASF. These plasmids, as well as empty plasmid (negative control) were transformed into *E*. *coli* BL21 and grown on Luria Broth (LB) plates with kanamycin (50 μg/mL) at 37°C. Individual colonies were picked and grown shaking in 5–10 mL culture of LB with 50 μg/mL kanamycin at 37°C until reaching OD600 ~0.6. Isopropyl β-D-1-thiogalactopyranoside (IPTG) was then added to 100 μM and incubation continued at 20°C for 16 hours with shaking for the induction of Cry14A proteins (or empty vector). The cultures were then pelleted and resuspended in S medium to an OD600 = 2. Dilution between empty vector and Cry14A-expressing cells was used for dose-response studies (*e*.*g*., 50% Cry14Aa = 1:1 Cry14Aa with empty vector, both OD = 2).

### Conditional EMS mutagenesis, screening, and subsequent outcrossing

Large non-condition screens for Cry14Aa resistant mutants (total ~794,000 mutagenized F2) was carried out as described for Cry5Ba [15]. Only two weakly resistant animals, both harboring new mutations in *bre-3* (confirmed by complementation testing and DNA sequencing) were identified. For the conditional screen, a synchronized population of L4 *bre-4*(*ye13*) hermaphrodites grown at 15° C was exposed to 0.5 mM ethyl methanesulfonate (EMS) for 4 hr. on a rocker at room temperature, washed 4 times with M9, re-plated onto NG plates, and propagated at 15°C for two generations, with bleaching at each generation to synchronize the population. At the F2 stage, 175,000 hermaphrodites (4% of the total F2 hermaphrodites) were shifted to 25°C on 100 mm enriched nematode growth medium (ENG) plates containing IPTG (500 μM) and kanamycin (50 μg/mL) (ENG-IK) spread with 20% Cry14Aa (diluted as above, grown on plates for 2 days at 20°C). *bre-4(ye13)* animals are not resistant to Cry14Aa at this concentration. The plates with 3000 L4 mutagenized hermaphrodites per plate were incubated for 48 hrs. at 25°C. Healthy looking hermaphrodites were transferred individually onto OP50 NG plates at 15°C, allowed to propagate, and tested multiple times on 60 mm ENG-IK plates with 20% Cry14Aa. Only one mutant reproducibly tested as resistant, *bre-4(ye13)*;*bre-6(ye123)*.

*bre-4(ye13);bre-6*(*ye123*) hermaphrodites were outcrossed as follows. *bre-4(ye13);GFP* males were crossed with *bre-4(ye13);bre-6*(*ye123*) hermaphrodites. GFP+ cross progeny (F1) were identified and allowed to self. Individual F2s were picked onto separate plates and grown until the F3. From the F3, seven L4s were picked and tested on 50% Cry14Aa in a liquid well assay (see below; 50% is a dose which readily discriminates between GFP-N2, *bre-4(ye13)* and *bre-4(ye13);bre-6(ye123)* hermaphrodites). Only wells with 100% alive hermaphrodites were selected as homozygous for *bre-4(ye13);bre-6(ye123)*, which was then confirmed with a dose-response assay. This procedure was repeated three more times for a total of four outcrosses. *bre-4(ye13)* was removed from this 4X outcross strain by crossing N2 males into *bre-4(ye13);bre-6(ye123)* hermaphrodites. We then sequenced individual resistant F2 lines for lack of the mutation present in *bre-4(ye13)*[25].

### Microscopy and imaging

Fourth staged larvae of *C*. *elegans* were seeded on ENG-IK plates spread with BL21 *E*. *coli* expressing Cry14Aa (100%) and incubated at 25°C for 48 h. Multiple worms were randomly picked from each of the different treatment conditions for image collection. Pictures were taken using ImageJ connected to an Olympus SZ-CTV compound microscope with an SZ60 objective.

### Mortality assays with Cry proteins

Cry proteins expression were induced as described above. In 48-well format, 40ul bacteria of OD600 = 2 at the stated concentration (*e*.*g*., 50% Cry14Aa or Cry14Ab) were combined with 150 μl S-medium and 5 μl 8 mM FUDR (5-fluoro-2′-deoxyuridine) to prevent the production of progeny that would complicate the assay[45]. Thirty to forty L4 hermaphrodites in 5 μL were pipetted into each well of 48-well plates at 25°C unless otherwise specified. To select L4 hermaphrodites, nematodes were grown based on L4 morphology not time, *e*.*g*., starting at the L1 stage, N2 animals were grown at 15°C for 56 hr. whereas *bre-6(ye123)* were grown for 72 hr. to achieve the same stage. Three technical replicates were used for each concentration, which was then repeated for a total of at least three independent experimental replicates. After six days the hermaphrodites in each well were transferred to glass counting well plates and prodded with an eyelash. Hermaphrodites were considered alive if they moved by poking or dead if they did not.

### Developmental inhibition assay

Wells in a 48-well format were prepared as above (including three technical replicates), except synchronized L1 hermaphrodites (30–40) were pipetted in each well and incubated at 25°C for 3 days. Hermaphrodites were moved to glass spot wells and imaged. Hermaphrodite growth was assessed by measuring the length of clearly separated, individual worms (worms overlapping on image collection were not measured) using Image J (version 1.46r). Each experiment was independently repeated three times.

### Characterization of fitness cost of worm

Growth rate assay (Fig 3A)–L1 stage nematodes (~30/plate) from N2 and *bre-6(ye123)* were pipetted onto three plates and incubated at 15°C or 25°C. Growth proceeded synchronously on each plate. The time until the first eggs appeared on each plate was noted to measure the time from the L1 stage to egg-bearing adult stage. This was normalized to N2 for each temperature. The results for this assay include three independent replicates.

Brood size assay (Fig 3B)–L4 staged nematodes (~1/plate) from N2 and *bre-6(ye123)* were picked one each to four 60 mm NG plates spread with *E*. *coli* OP50 and incubated at 15°C or 25°C for 24-hour periods. After each period, the original adult nematodes was picked to a new plate. Progeny from the original parent nematodes were allowed to grow an additional 24 hour at the same temperature before they were counted. This process was continued every 24 hour until the original adult nematodes ceased to produce additional progeny. The results for this assay include three independent replicates. The relative brood sizes were obtained by normalizing to N2 for each temperature. For S1 Table (resistance of Cry5Ba glycosphingolipid receptor mutants to Cry14Aa and Cry14Ab), brood size assays were performed as described [14,26] except that instead of dosing μg/mL, OD normalized bacteria were diluted with vector control such that 0% is all vector only *E*. *coli* and 100% is undiluted Cry14A-expressing *E*. *coli*.

### Whole genomic sequencing

To identify *bre-6* gene, we used the variant discovery method [31]. *bre-4*(*ye13*);bre-6 (*ye123*) outcrossed hermaphrodites were outcrossed as above to generate a new generation of homozygous mutants. 96 F2 homozygous *bre-4(ye13);bre-6(ye123)* re-segregant lines were phenotypically identified. After harvesting and mixing the equal number of worms from the 96 lines in M9 buffer, the worm pellet was washed three times with M9 buffer to get rid of the gut bacteria and frozen. These pellets were sent to Genewiz, Inc. DNA preparation, sequencing, and data analysis were performed by Genewiz Inc. (Massachusetts, USA). Briefly, genomic DNA was extracted using the Qiagen QIAmp DNA Kit and HT DNA Kit (Qiagen, Hilden, Germany) and prepared using NEBNext Ultra DNA Library Prep Kit. After clustering, the samples were loaded on the Illumina HiSeq instrument and sequenced using a 2x 150 paired-end (PE) configuration. The trimmed reads for samples *bre-4*;GFP, *bre-4 (ye13);bre-6 (ye123)* were then mapped according to the reference genome for *C*. *elegans* from NCBI. Then SNPs/INDELs were detected using the Fixed ploidy variant detection model within the CLC Genomics Workbench software, version 10.0.1 (https://www.qiagenbioinformatics.com/). A list of variants was detected in the samples: *bre-4* (*ye13*);GFP, *bre-4* (*ye13*);*bre-6* (*ye123*). Genewiz additionally filtered *bre-4*(*ye13*);*bre-6*(*ye123*) for different standard variants when compared to *bre-4* (*ye13*);GFP samples in CLC Genomics Workbench 10. In the initial comparisons, a mutation in *vha-5* was also identified as segregating with the resistance phenotype (99.8%;*vha-5* is in the same pathway as *nhr-31* and is closely linked to *nhr-31*). However, this mutation was lost upon outcrossing to remove of *bre-4(ye13)* and is not present in any of the data here except for Fig 1A.

### Extrachromosomal transgenic array rescue

For the rescue experiments, germline transformation was performed as described (25). *nhr-31* complete genomic DNA was obtained by PCR amplification of *nhr-31* gene and 2 kb 5’ promoter region and 1 kb 3’ untranslated region (UTR) of the gene. Forward primer sequence was 5’-CAACTTCAAGCCTCGTGTACC-3’ and reverse primer sequence was 5’-GTGTTGTCTCCATGTGAGAAAGC-3’. The constructs were verified by sequencing using a primer (5’-ATG CGA GGA GAT TTA AGG ACA AGC -3’). Column-purified PCR DNA (30–100 ng/μl) was co-injected with pRF4::rol-6(su1006) (a dominant allele conferring a roller phenotype) plasmid (100 ng/μl) as an injection marker (32). Two independent lines of each transgenic strain were examined.

### RNA inhibition (RNAi)

RNAi experiments were performed by feeding worms *E*.*coli* strain HT115 (DE3) transformed with the control vector L4440 or L4440 subcloned with a gene fragment of *nhr-31*. *Nhr-31* genomic fragment was amplified by primers (5′-ACGCTAATACTTCATCCAAA-3′ and 5′-GCTGATTACGAGAAATTTCA-3′) and cloned into L4440. Single colonies of bacteria were picked and grown in small culture LB tubes with antibiotic (100 μg/mL ampicillin) overnight at 37°C and seeded onto NG plates supplemented with 1mM IPTG and ampicillin (100 μg/mL). The plates were dried overnight at room temperature, to which L1 larvae of N2 hermaphrodites were seeded. When the larvae became L4 they were transferred into the wells for the mortality assay. *nhr-31* knock-down in these experiments was confirmed by real-time PCR (forward primer: 5’-ATG CGA GGA GAT TTA AGG ACA AGC -3’, reverse primer: CCA TCC GTC GAC CAT CTA ATG CAG-3’), which demonstrated a 94% reduction in *nhr-31* mRNA levels. The mortality assay was performed in wells with 50% L4440 expressing *nhr-31* RNAi and 50% BL21 expressing Cry14Aa.

### Gene editing by Crispr

A vector expressing *rol-6(su1006)* was used as a co-injection marker (32). A deletion mutant *nhr-31(ye127)* was generated by injecting pre-assembled Cas9 ribonucleoprotein complex using oligos as template as described [46]. Modifications and genome editing events were identified by sequencing ~400 nucleotides of DNA from the F2 progeny of F1 rollers in this region, confirming that the same mutation as in *nhr-31(ye123)* was recreated.

### RNAseq

N2 and *bre-6 (ye123)* hermaphrodites at the fourth larval stage were seeded onto ENG-IK plates spread with BL21 *E*. *coli* containing empty IPTG-inducible pRSF vector or pRSF with Cry14Aa incubated at 25°C for 4 hrs. Worms were harvested and washed with M9 and snap-frozen and stored at -80°C for total RNA isolation. After the isolation of total RNA using RNeasy mini kit (Qiagen), a complementary DNA library was prepared and sequencing was performed according to the Illumina standard protocol by Beijing Novel Bioinformatics Co., Ltd. (https://en.novogene.com/). RNA-Seq reads were aligned to *C*. *elegans* genome assembly Ce11 (WS245) using STAR Aligner v2.5.2a_modified. Gene expression was quantified using RSEM v1.2.29 (rsem-calculate-expression). Transcripts Per Million (TPM) values calculated by RSEM were used in differential expression analysis of genes of interest. TPM values from 3 replicates per condition were tested using Graphpad Prism multiple t-tests. Multiple testing adjustment was performed using two-stage step up method of Benjamini, Krieger and Yekutieli with a desired FDR value of 0.01.

### Data plotting and Statistical analyses

All the data were plotted and statistics analyzed with Prism 10 (GraphPad Software, California, USA). At least 3 independent toxicity assays were combined to plot the graph. Statistical significances were determined as indicated in figure legends except for RNAseq (see above). For S1 Table, IC_50_ values were calculated using PROBIT (from XLSTAT add-on to EXCEL, Addinsoft;[43]). All mean and standard error values used to plot the graphs are included in S1 Data.

## Supporting information

S1 Fig *C*. *elegans* after 2 days of plating on vector-only (0%) or Cry14Aa (100%)-expressing *E*. *coli*. All pictures were taken at the same magnification. Relative to 0% control, the *bre-4(ye13);bre-6(ye123)* hermaphrodites were larger and were more viable on Cry14Aa than N2 or *bre-4(ye13)* hermaphrodites. Scale bar = 0.5 mm.
(TIF)

S1 Table Resistance of Cry5Ba glycosphingolipid receptor mutants to Cry14Aa and Cry14Ab based on brood size. IC_50_ (%) is the inhibitory concentration (% Cry14A bacteria) that leads to 50% reduction in progeny production in that mutant relative to vector-only control. 95% Cl (%) is the 95% confidence interval for the IC_50_. Resistance ratio is the IC_50_(*bre* mutant) / IC_50_(N2).
(XLSX)

S1 Data Means and standard error values for all figures. (XLSX)
