# Supplementary figures and images for: Resistance to Cry14A family Bacillus thuringiensis crystal proteins in Caenornabditis elegans operates via the nhr-31 transcription factor and vacuolar-type ATPase pathway
Source: PLoS Pathog. 2024 Oct 18;20(10):e1012611. doi: 10.1371/journal.ppat.1012611 (PMC11524453; doi:10.1371/journal.ppat.1012611)

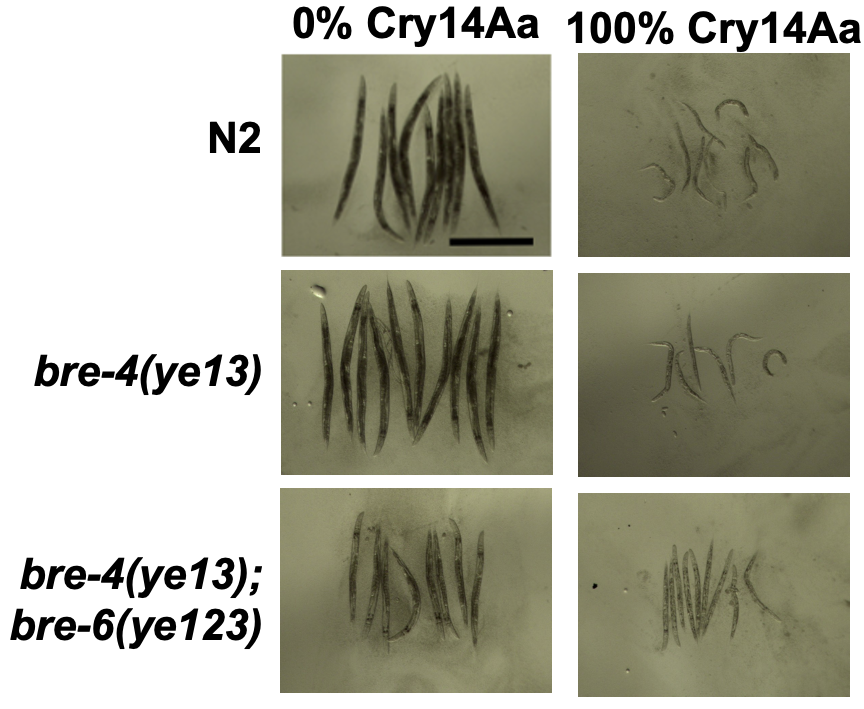

Supplement: S1 Fig — All pictures were taken at the same magnification. Relative to 0% control, the bre-4(ye13);bre-6(ye123) hermaphrodites were larger and were more viable on Cry14Aa than N2 or bre-4(ye13) hermaphrodites. Scale bar = 0.5 mm. (TIF) [file ppat.1012611.s001.tif]
